# Detection of Degenerative Changes on MR Images of the Lumbar Spine with a Convolutional Neural Network: A Feasibility Study

**DOI:** 10.3390/diagnostics11050902

**Published:** 2021-05-19

**Authors:** Nils Christian Lehnen, Robert Haase, Jennifer Faber, Theodor Rüber, Hartmut Vatter, Alexander Radbruch, Frederic Carsten Schmeel

**Affiliations:** 1 Department of Neuroradiology, University Hospital Bonn, Rheinische Friedrich-Wilhelms-Universität Bonn, 53127 Bonn, Germany; nils.lehnen@ukbonn.de (N.C.L.); robert.haase@ukbonn.de (R.H.); alexander.radbruch@ukbonn.de (A.R.); 2 Research Group Clinical Neuroimaging, German Center for Neurodegenerative Diseases, 53127 Bonn, Germany; 3 Department of Neurology, University Hospital Bonn, Rheinische Friedrich-Wilhelms-Universität Bonn, 53127 Bonn, Germany; jennifer.faber@ukbonn.de; 4 Department of Epileptology, University Hospital Bonn, Rheinische Friedrich-Wilhelms-Universität Bonn, 53127 Bonn, Germany; theodor.rueber@ukbonn.de; 5 Department of Neurosurgery, University Hospital Bonn, Rheinische Friedrich-Wilhelms-Universität Bonn, 53127 Bonn, Germany; Hartmut.vatter@ukbonn.de

**Keywords:** deep learning, lumbar spine, MRI, automated reading, diagnostic performance, disc protrusion, disc bulging, spinal canal stenosis, nerve root compression, spondylolisthesis

## Abstract

Our objective was to evaluate the diagnostic performance of a convolutional neural network (CNN) trained on multiple MR imaging features of the lumbar spine, to detect a variety of different degenerative changes of the lumbar spine. One hundred and forty-six consecutive patients underwent routine clinical MRI of the lumbar spine including T2-weighted imaging and were retrospectively analyzed using a CNN for detection and labeling of vertebrae, disc segments, as well as presence of disc herniation, disc bulging, spinal canal stenosis, nerve root compression, and spondylolisthesis. The assessment of a radiologist served as the diagnostic reference standard. We assessed the CNN’s diagnostic accuracy and consistency using confusion matrices and McNemar’s test. In our data, 77 disc herniations (thereof 46 further classified as extrusions), 133 disc bulgings, 35 spinal canal stenoses, 59 nerve root compressions, and 20 segments with spondylolisthesis were present in a total of 888 lumbar spine segments. The CNN yielded a perfect accuracy score for intervertebral disc detection and labeling (100%), and moderate to high diagnostic accuracy for the detection of disc herniations (87%; 95% CI: 0.84, 0.89), extrusions (86%; 95% CI: 0.84, 0.89), bulgings (76%; 95% CI: 0.73, 0.78), spinal canal stenoses (98%; 95% CI: 0.97, 0.99), nerve root compressions (91%; 95% CI: 0.89, 0.92), and spondylolisthesis (87.61%; 95% CI: 85.26, 89.21), respectively. Our data suggest that automatic diagnosis of multiple different degenerative changes of the lumbar spine is feasible using a single comprehensive CNN. The CNN provides high diagnostic accuracy for intervertebral disc labeling and detection of clinically relevant degenerative changes such as spinal canal stenosis and disc extrusion of the lumbar spine.

## 1. Introduction

Lower back pain is among the leading causes of morbidity and disability, with an increasing prevalence due to the steadily aging population worldwide [1]. According to the American College of Radiologists, lumbar spine magnetic resonance imaging (MRI) is the preferred imaging modality to rule out causes of complicated lower back pain and to decide whether conservative or invasive therapeutic approaches should be considered [2,3]. Subsequently, the number of MRI studies of the lumbar spine has been rising over the last decades at a much higher rate than the number of trained radiologists who could adequately interpret the MR images [4]. To address this challenge, automated systems for pathology detection and grading on MRI scans can be utilized to support clinical reporting and reduce the steadily increasing workload for radiologists.

Machine learning algorithms to analyze lumbar spine imaging have successfully been used for segmentation, single measurements, and labeling tasks in radiographs [5,6], computed tomography (CT) [7,8,9,10], and MRI studies of the spine [11,12,13,14,15,16]. To date, a few attempts have been made to perform multiple automated radiological gradings such as Pfirrman grading, disc narrowing, spondylolisthesis, central canal stenosis, endplate defects, and bone marrow alterations with a single software solution [17]. However, to the best of our knowledge, there are no software solutions available yet that address the detection of multiple pathologies in MR imaging studies of the lumbar spine. Here, we evaluated the automated detection of degenerative changes in the lumbar spine using a comprehensive software solution (“CoLumbo”, SmartSoft Ltd., Varna, Bulgaria). This algorithm is designed to label the segments of the lumbar spine and to detect a broad variety of degenerative pathologies based on a convolutional neural network (CNN). CNNs have been widely used in the field of medical imaging. They are known to be efficient and accurate, usually outperforming other machine learning, or more specifically, deep-learning-based approaches not only for medical imaging analysis [18,19,20]. CNNs are space invariant networks. Based on the fact that the response for a shifted image should be a similar shifted feature map, they use a shared-weight architecture. Generally, convolution filters (for example a 3 × 3 matrix) slide along input features and subsequently provide a corresponding output feature map. This methodology can significantly reduce the number of input parameters when compared to standard neural networks, and hence reduces the need for large data sets [21]. Moreover, from a practical point of view, CNNs are more straightforward to train than recurrent neural networks, as the latter face issues like exploding or vanishing gradient [22,23]. As things stand at present, the leading software solutions in the general imaging segmentation challenges and spine segmentation challenges are currently based on CNNs. Specifically, CoLumbo aims to detect the presence and the location of disc herniation, disc bulging, nerve root compression, spinal canal stenosis, and spondylolisthesis. Currently, this CNN-based algorithm is leading the IVDM3Seg challenge on automatic intervertebral disc localization and segmentation from 3D multimodality MR (M3) images (IVDM3Seg, entry smartsoftv2) spine segmentation competition [24] which has been established in association with the international conference on Medical Image Computation and Computer Assisted Intervention (MICCAI) 2018, Granada, Spain.

The primary aim of this study was to validate the algorithm’s diagnostic performance and to determine whether the automated approach is generally feasible and provides the potential for clinical use. Therefore, we evaluated the diagnostic accuracy of the findings detected by the algorithm and the generalizability of the CNN to a new previously unseen data set.

## 2. Materials and Methods

Institutional Review Board approval was obtained for this retrospective diagnostic study and the need for written informed consent was waived.

### 2.1. Case Selection and Expert Reading

Inclusion criteria were the following: patients aged between 18 and 70 years undergoing routine MRI of the lumbar spine due to lower back pain were eligible to participate in this study. Exclusion criteria were presence of vertebral fractures and/or active inflammation as determined on routine clinical MRI, history of previous or concomitant malignancy, prior spine surgery, and metallic implants on the spinal level. Additionally, patients with incomplete examinations or severe motion and/or susceptibility artifacts were excluded. In patients who underwent repeated MRI scans, only the first examination was included in the analysis to avoid inclusion of repeated observations made in the same patient. The MR images included in the study were evaluated retrospectively by an experienced radiologist (with 5 years of experience in interpreting spine MRI) under full consideration of the originally written reports and the clinical history of the patients. This expert reading served as the diagnostic reference standard. The expert reader analyzed a total of 888 lumbar spine segments (TH12/L1-L5/S1) for the presence or absence of disc herniation, spinal canal stenosis, and bulging. For nerve root compression, 1036 segments (TH12-S1) were analyzed. Disc herniation was further subdivided into protrusion and extrusion in order to differentiate smaller and potentially less relevant herniations from larger and clinically more relevant findings. Disc extrusion was defined as a focal herniation with the distance between the edges of the herniating disc material into the spinal canal being greater than the diameter at the base of the herniation. Disc bulging was defined as the annulus fibrosus extending beyond the edges of the disc space, affecting more than 25% of the circumference of the disc. These classifications were done according to the nomenclature proposed by the consensus statement of the American Society of Spine Radiology (ASSR), American Society of Neuroradiology (ASNR), and North American Spine Society (NASS) [25]. Listhesis was graded according to the Meyerding classification [26]. Spinal canal stenosis was deemed present when there was a loss of anterior CSF signal on the sagittal images and a loss of CSF signal in the spinal canal on the axial images with an aggregation of the cauda equina fibers, according to moderate or severe spinal canal stenosis as proposed by Lee et al. [27]. 

### 2.2. MR Imaging Protocol

All lumbar spine MR examinations were performed using either a clinical 3.0T MR scanner (Achieva, Philips Healthcare, Best, The Netherlands) or a 1.5 T MR scanner (Achieva, Philips Healthcare, Best, The Netherlands). All patients were placed in the supine position. MR imaging of the spine was acquired according to the routine clinical MRI protocol used at our institution which included at least a sagittal T1-weighted spin-echo (450–750/6–12 [repetition time (TR) msec/echo time (TE) msec]), a sagittal and axial T2-weighted turbo spin-echo sequence (3000–5000/80–120 [TR/TE]) as well as a sagittal T2 spectral attenuated inversion recovery (SPAIR)-weighted turbo spin-echo sequence (3000–5000/50–120 [TR/TE]) or T2-weighted mDixon sequence (3000–5000/50–120 [TR/TE]). Slice thickness was 4 mm for sagittal imaging (slice gap 0.4 mm) and 3.5 mm for axial imaging (slice gap 0.35 mm). Field of view and matrix size were tailored to the individual patients’ characteristics by the radiological technician. T2-weighted axial and sagittal images of the 146 patients were anonymized and extracted from the institute’s archive as DICOM files to make them accessible to the algorithm for image analysis. 

### 2.3. Machine Learning Algorithm and Image Analysis

The software automatically registers anatomic structures of the lumbar spine like vertebral bodies, intervertebral discs, the pedicles, the spinous processes and laminae, the flava ligaments, the dural sac, the nerve roots, the aorta to measure its diameter, and the erector spinae muscle to analyze its fatty degeneration. Additionally, the CNN is designed to detect multiple pathologies, like disc herniation and disc bulging, spinal canal stenosis, nerve root compression, and spondylolisthesis.

**Figure 1:**
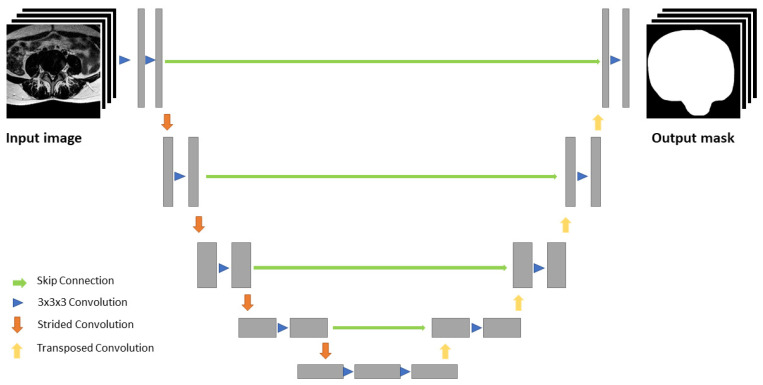
U-Net-like segmentation architecture. The input image is first downscaled in four steps, then the low-resolution feature map is upscaled to the resolution of the input image. Additional feature maps from the downscaling part are incorporated at each upscaling layer.

The algorithm in this study incorporates a three-step process for pathology identification and characterization: 1. segmentation of different tissue types; 2. measurements of clinically used distances observable in the image; 3. diagnosis, which is described in detail elsewhere [28]. In brief, a 2D single modality algorithm using a U-Net-based convolutional neural network (CNN) [29] is utilized in each plane of view. Each of the four up-/downsampling steps is achieved using 2 × 2 upsampling/maxpool operations. Additional feature maps from the downscaling part are incorporated at each upscaling layer. These feature maps are based on a fully convolutional network, similar to ResNet-50 [30]. The initial low-resolution feature map (64 × 64) is then upscaled using a U-Net-like architecture to the resolution of the input image (512 × 512) (Figure 1). In this way, higher-level, lower-resolution classification features are used as context and the higher resolution is used for finer details.

As a next step, the segmentation is utilized to perform measurements and to classify the different pathologies. For disc herniation and disc bulging, the segmented contour of the vertebral bodies adjacent to the intervertebral disc is projected over the intervertebral disc on the axial slice at disc level. The algorithm then identifies the parts of the disc that exceed the projection of the contour of the neighboring vertebral bodies and the anterior-posterior distance of the disc exceeding the projected contour of the vertebral bodies is measured. Herniation or bulging are defined as present when the diameter exceeds 3 mm by default. The differentiation between disc herniation and disc bulging is based on the lumbar disc nomenclature: version 2.0 [25].

For spinal canal stenosis, the cross sectional area of the dural sac is measured. A dural sac area of 75 to 100 mm^2^ is defined as relative stenosis, a dural sac area of less than 75 mm^2^ is defined as absolute stenosis by the developers. Both relative and absolute spinal canal stenosis reported by the software were counted as positive findings by the authors.

Nerve root compression is reported as such, when a herniated disc or other tissues are in contact with the nerve root and the nerve root is deviated. For deviation of the nerve root, the algorithm calculates where the nerve root with contact to a herniated disc is supposed to be according to the position of the nerve root on the adjacent axial slices.

For spondylolisthesis, a tangent through the posterior aspect of the vertebral bodies adjacent to the specific intervertebral disc is drawn. These tangents cross a line drawn along the superior endplate of the inferior vertebral body at different points. The distance between these points is measured and compared to the length of the superior endplate of the inferior vertebral body and the ratio of the two distances is calculated, determining the percentage of spondylolisthesis. Spondylolisthesis is graded according to the Meyerding classification.

**Figure 2:**
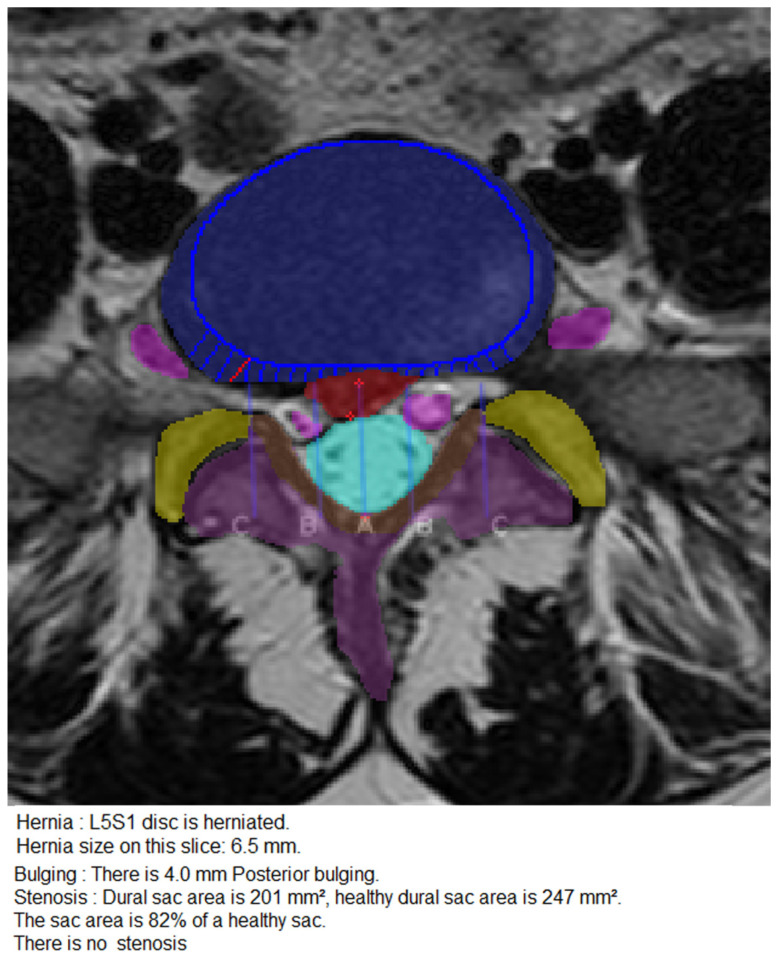
Measurement and classification of disc herniation, disc bulging, nerve root compression and spinal canal stenosis. T2-weighted, axial slice through the segment L5/S1 at disc level showing disc herniation, disc bulging, no nerve root compression and no spinal canal stenosis. The projected contour of the vertebral body adjacent to the disc is represented by the rounded blue line, the intervertebral disc is represented by the blue area. The red area represents herniated disc material, the distance between the red crosses is measured 6.5 mm and is therefore correctly classified as disc herniation. The blue lines and the single red line on the right side of the disc perpendicular to the projection of the contour of the vertebral body represent measurements of the disc exceeding the boundaries of the adjacent vertebral bodies, correctly reported as 4 mm bulging. The nerve roots (pink) have no contact to either the herniated or the bulging parts of the disc, therefore nerve root compression was correctly classified as absent. The light blue area represents the dural sac of 201 mm^2^. There was no spinal canal stenosis reported.

The process of measurement and classification of disc herniation, disc bulging, nerve root compression and spinal canal stenosis is illustrated in Figure 2.

The initial training set consisted of 1500 patient studies, including a total number of 20,000 axial slices and 10,000 sagittal slices manually annotated by board-certified radiologists. These patient studies were provided by three different European medical centers acquired at two different 1.5 T clinical MRI scanners (GE Medical Systems Signa HDxtm, GE Healthcare, Chicago, IL, USA; Siemens Verio, Siemens Healthineers, Erlangen, Germany). The characteristics of the slices in the dataset varied: Voxel thickness: 1 to 10 mm. Repetition time: 940 to 6739 ms. Echo time: 60 to 300 ms. Axial resolutions: 192 × 192, 512 × 512. Sagittal resolutions: 512 × 768, 256 × 280. The axial slices were aligned parallel to the intervertebral disc. The primary development and implementation including training and validation of the CNN were performed by SmartSoft Ltd., Varna, Bulgaria. 

The training started with a model that had a feature extractor pretrained on the COCO dataset [31] while the rest of the model was trained without pretraining. For the segmentations, pixelwise cross-entropy loss was used. The optimization algorithm was Gradient Descent with momentum. L2 regularization, dropout and data augmentation were used. The training process lasted for 300,000 epochs.

Each MRI study was analyzed by the algorithm, thereby segmented and labeled for each lumbar spine segment and subsequently classified for presence or absence of the previously mentioned pathologies. The results were taken from an automatically generated report on the software user interface and compared to the findings detected by the expert reader.

### 2.4. Statistical Analysis

Statistical analyses were performed with R version 4.0.3 and RStudio version 1.2.5033 (RStudio, Inc., Boston, MA, USA) using the caret package [32]. All applicable clinical and imaging data are given as mean ± standard deviation, unless otherwise specified. Statistical significance level was set at *p* < 0.05. The diagnostic performance of the CNN was compared to the radiologist’s findings using confusion matrices and the McNemar test. Additionally, we calculated sensitivity, specificity, positive predictive value (PPV), negative predictive value (NPV), and accuracy.

## 3. Results

### 3.1. Patient Cohort

Between November 2018 and July 2020, 567 consecutive patients with lower back pain underwent MRI examinations of the lumbar spine at our institution, 421 of whom were excluded from study enrollment based on the exclusion criteria stated above. The remaining patient cohort consisted of 146 subjects, 81 males (55.5%) and 65 females (44.5%). Mean age was 48.7 years (median 49.5 years, range 19–70 years). An additional subset analysis revealed no statistically significant differences regarding both gender and age distribution between the patient groups examined at 1.5 T (59 men, 47 females; mean age, 48.36 years, range 19–70 years) and 3 T (22 men, 18 females; mean age, 49.55 years, range 21–69 years). In a total of 888 segments analyzed, expert reading detected 77 disc herniations (8.7%) out of which 46 were labelled as disc extrusions (5.2%), 133 disc bulgings (15.0%), 35 spinal canal stenosis (3.9%), and 20 spondylolistheses (2.3%). For nerve root compression, 59 nerve root compressions (5.7%) were found in the 1036 segments analyzed. All spondylolistheses detected by the radiologist were reported as grade I according to the Meyerding classification.

### 3.2. CNN Diagnostic Performance

Mean processing time for each individual MRI study was 9:25 min (range, 8:34 to 11:20 min). All imaging studies could successfully be processed by the software.

The CNN detected a total of 156 disc herniations (17.6%), 222 disc bulgings (25.0%), 35 spinal canal stenosis (3.9%), 123 nerve root compressions (11.9%) and 122 spondylolistheses (13.7%). All spondyolistheses detected by the CNN were reported as grade I according to the Meyerding classification.

**Figure 3:**
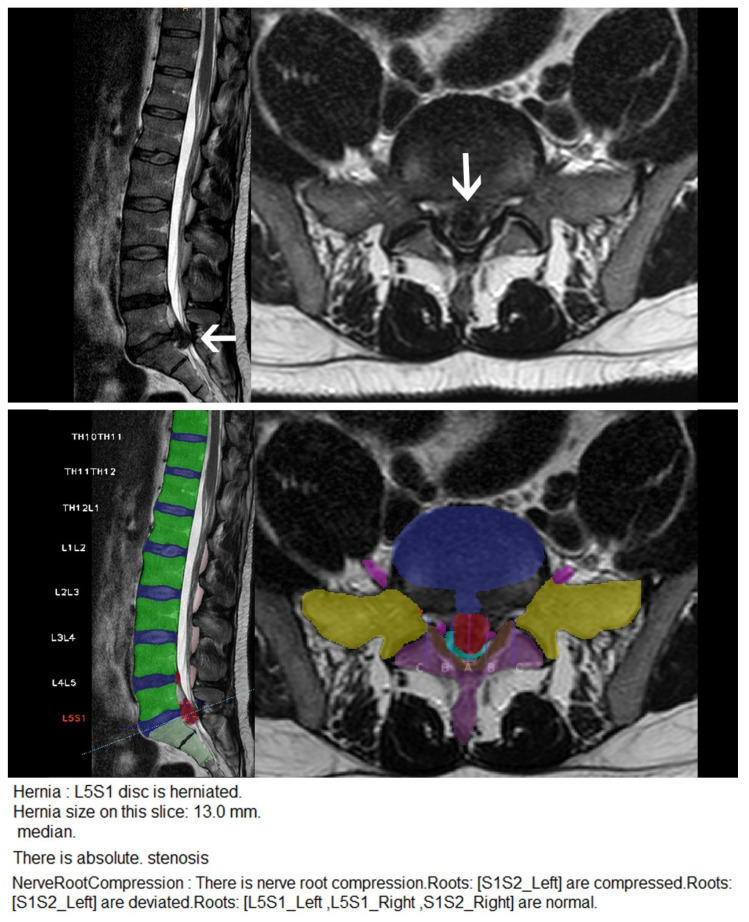
Disc extrusion and spinal canal stenosis correctly detected by the CNN. Upper row: Sagittal and axial T2 weighted MR images of the lumbar spine showing a large disc extrusion (arrows) with severe spinal canal stenosis. Middle row: User interface of the CNN. Each segment of the lumbar spine is correctly labelled by the CNN with the segment showing the most severe pathology being highlighted in red. The vertebral bodies are highlighted in green; the lumbar discs are highlighted in blue. The transverse processes are highlighted in yellow; the laminae and the spinous processes are highlighted in purple; the flava ligaments are highlighted in brown. The dural sac is highlighted in light blue, the nerve roots are highlighted in pink. Disc bulgings and disc herniations are highlighted in red. Lower row: Excerpt of the written report automatically generated by the software.

**Figure 4:**
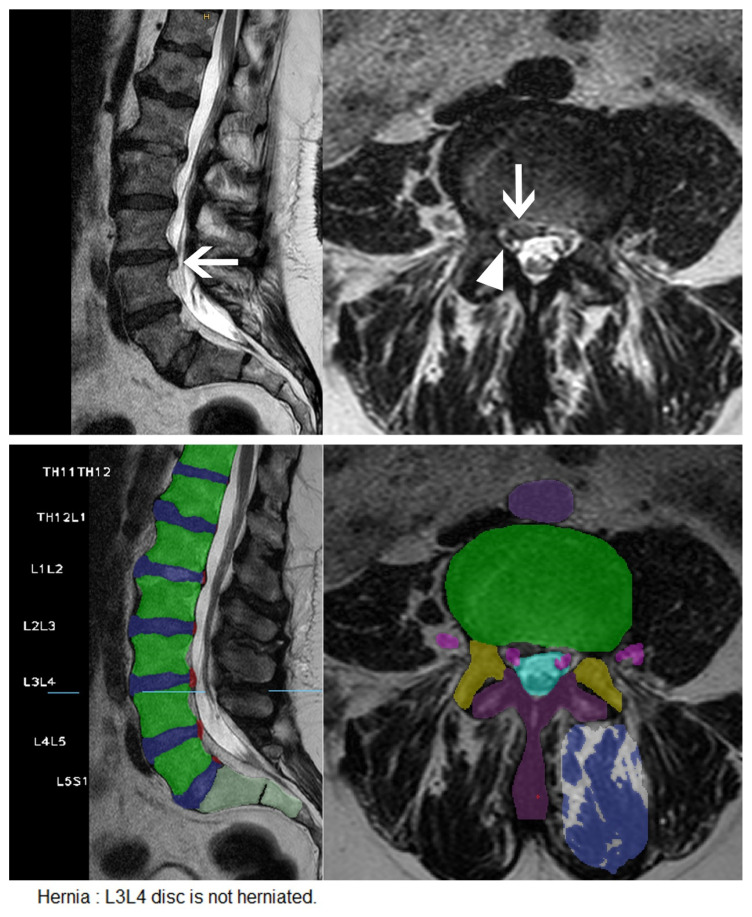
Small disc extrusion with nerve root compression missed by the CNN. Upper row: Sagittal and axial T2 weighted images of the lumbar spine showing a small disc extrusion at the level of L3/L4 on the right side (arrows) with compression of the nerve root L3 on the right side (arrowhead). Middle row: User interface of the CNN with correct identification of the nerve roots (pink), but without detection of the small disc herniation on the right. Lower row: Excerpt from the written report automatically generated by the software.

**Figure 5:**
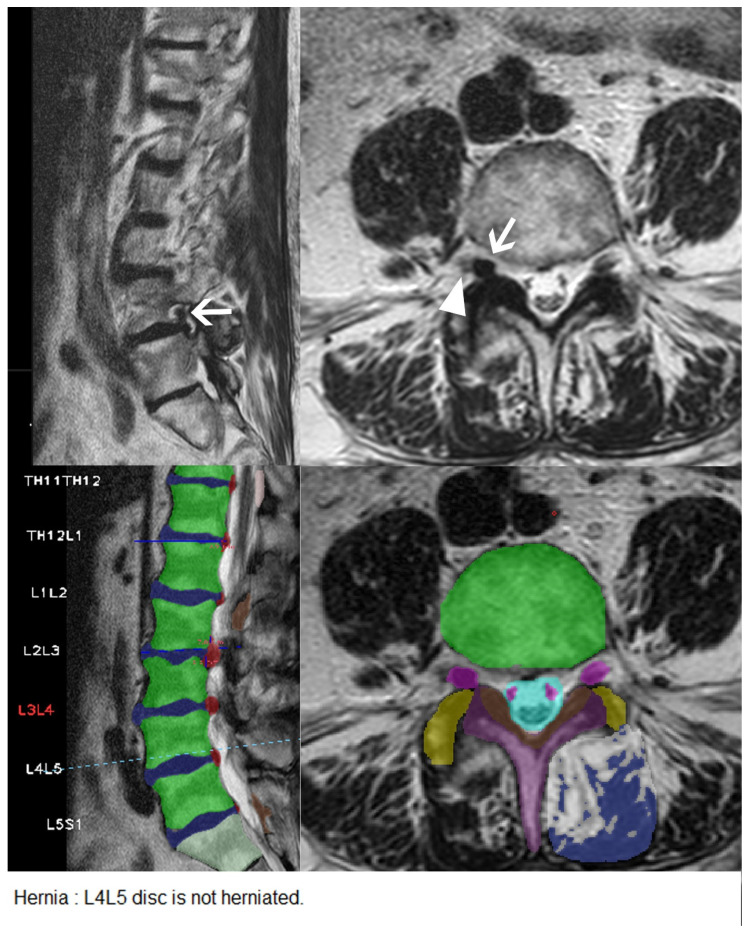
Intraforaminal disc extrusion missed by the CNN. Upper row: Sagittal and axial T2 weighted images of the lumbar spine showing an intraforaminal disc extrusion at the level of L4/L5 on the right side (arrows) with compression of the nerve root L4 on the right side (arrowhead). Middle row: User interface of the CNN. The CNN misinterpreted the herniated disc material as the nerve root L4, highlighted in pink. The actual nerve root L4 is situated lateral to the herniated disc material and has not been identified by the algorithm. Lower row: Excerpt from the written report automatically generated by the software.

Results are summarized in Table 1. Examples of accurately and inaccurately classified findings by the algorithm are shown in Figure 3, Figure 4 and Figure 5.

For vertebrae and segment detection and labeling, the CNN correctly identified all lumbar spine segments in our cohort. Concordant classification between the expert reader and the CNN was observed in 771 of the 888 segments for disc herniation, with 58 (7.5%) being true positives. Discordant classification was observed in 117 of the 888 segments for disc herniation, with 19 of these 117 segments being false negatives. Out of 46 disc herniations that were further classified as disc extrusions by the radiologist, 41 were detected by the algorithm. For disc bulging, concordant classification between the reader and the CNN was observed in 671 of the 888 segments, with 69 (10.3%) of the 671 segments being true positive findings. Discordant classification for disc bulging was observed in 217 segments, with 64 of these segments being false negatives. For nerve root compression, concordant classification was observed in 938 of the 1036 spine segments, with 42 (4.5%) of the 938 segments being true positives. Discordant classification for nerve root compression was observed in 98 segments with 17 of these segments being false negatives. For spinal canal stenosis, concordant classification was observed in 871 of the 888 segments, with 27 (3.1%) of the 871 segments being true positives. Discordant classification was observed in 17 segments, out of which nine were false negatives. For spondylolisthesis, discordant classification was observed in 110 segments, with four listheses being present according to the radiologist but not reported by the CNN, and 106 listheses being present according to the CNN but not diagnosed by the radiologist.

The accuracy of the CNN was 100% for vertebrae detection and segment labeling (95% CI: 1; sensitivity 100%; specificity 100%, PPV 100%; NPV 100%; *p* = 1; data not shown in Table 1). The accuracies of the CNN were 86.8% for disc herniation (95% CI: 0.84, 0.89, *p* < 0.001), 86.5% for disc extrusion (95% CI: 0.84, 0.89, *p* < 0.001), 75.6% for disc bulging (95% CI: 0.73, 0.78, *p* < 0.001), 98.1% for spinal canal stenosis (95% CI: 0.97, 0.99, *p* = 1), 90.5% for nerve root compression (95% CI: 0.89, 0.92, *p* < 0.001), and 87.6% for spondylolisthesis (95% CI: 85.26, 89.21, *p* < 0.001), respectively.

Additional and dedicated subset analyses of our cohort scanned at 1.5 T and 3 T revealed no statistically significant differences regarding diagnostic performance and can be found in the Supplemental Materials in Tables S1 and S2.

## 4. Discussion

This single-center study compared the diagnostic performance of a single comprehensive CNN, trained on lumbar spine degenerative changes, with an expert radiologist’s reading for labeling spine segments and detecting disc herniations, nerve root compression, spinal canal stenosis, and spondylolisthesis on lumbar spine MR images in a randomly selected retrospective cohort. As mentioned earlier, the software is designed to solve three tasks: segmentation, measurements and diagnosis, while our study focused on the third task, diagnosis, to determine the clinical applicability, without analyzing the accuracy of the segmentation and the measurements in detail.

Our major findings revealed that, in a patient cohort with a wide age range, the CNN was highly consistent with the radiologist’s expert reading and yielded moderate to high diagnostic sensitivities and specificities for the detection of lumbar degenerative changes ranging from 52–89% and 80–99%, respectively. Owing to the high diagnostic accuracies and NPVs of the CNN, our data suggest that both clinical reading times and human input can possibly be reduced in lumbar MR imaging studies that were reported to be unsuspicious by the CNN. Our data also demonstrated promising results for the detection of both disc herniation and extrusion. Substantial disagreement between the expert reader and the CNN was found for disc bulging and spondylolisthesis. This might be due to the classification of even minor bulging by the automated methods, whereas the expert reader might tend to neglect clinically irrelevant findings, such as minor bulging that does not cause spinal stenosis or nerve root compression. The positive predictive value of only 13.11% for the detection of spondylolisthesis is partially due to the algorithm classifying a subset of segments as spondylolisthesis grade 0–1 according to the Meyerding classification. These segments were interpreted as spondylolisthesis grade 1 being present in the CNN analysis by the authors.

Most of the previous approaches mainly focused on automated detection and classification systems for a variety of individual spine pathologies. The automated detection methods used to date can be divided into three main types. The first type is the automatic localization of one or two types of spine structures, which are capable of depicting specific anatomical structures [33]. The second type comprises automatic segmentation of one or two types of spine structures [34], and the third type is simultaneous localization and segmentation of different spine structures [35]. These previously used detection methods have achieved accurate detection of one or two types of spine structures, but they have not been able to simultaneously perform radiological grading or diagnosis. Therefore, most AI-assisted radiological grading systems have been limited to one type of spinal structure, such as spondylolisthesis grading [36]. To date, there have been few attempts to simultaneously classify multiple pathologies on MR imaging studies of the lumbar spine to support diagnosis with a single comprehensive software solution. Recently, Jamaludin et al. trained an Oxford SpineNet software system, a machine-learning-based system for automatic analysis of T2-weighted spine MRI scans obtained from DICOM files. They reported that the system can automatically classify a variety of degenerative changes in the lumbar spine including Pfirrmann grade, disc stenosis, spondylolisthesis, central canal stenosis, Modic changes, and bone marrow changes. The software was able to classify multiple radiological features at the same time. However, a major drawback of this approach is that only the preprocessed disc volumes were used as inputs, so only one type of spinal structure could be analyzed. Another drawback of this approach was that only sagittal T2-weighted images could be analyzed, which particularly limited the ability to correctly classify spinal canal stenosis [17]. Moreover, automatic detection of disc herniation or nerve root compression was also not considered in that study, findings that must be considered when attempting to automate the reading of lumbar spine MRIs to reduce the radiologist’s workload in clinical practice. To our knowledge, our study is the first to externally validate a comprehensive software solution that focuses on the diagnosis of clinically relevant degenerative changes such as disc herniation and nerve root compression, in addition to the sole graduation of single degenerative changes. Our approach differs from most preliminary studies since it can process both axial and sagittal slices of lumbar MRIs, leading to markedly enhanced detection rates of spinal pathologies compared to the aforementioned study by Jamaludin et al. and a recent study by Won et al. (e.g., in case of spinal canal stenosis, our accuracy was 98% vs. 95% vs. 83%, respectively) [37], even though direct comparison of the methods on different data sets is methodologically difficult.

External validation is an important step in the validation of a deep-learning-based, predictive model to avoid overfitting to the training data set, potentially resulting in an overestimation of the model’s diagnostic performance [38,39]. Although various studies have shown that the diagnostic performance of AI algorithms can vary across different institutions, only 6% of studies that evaluated the diagnostic performance of AI algorithms performed external validation to ensure their generalizability [40,41]. The current study served as an external validation for the CNN that had been trained on 1500 data sets. Our results showed that the CNN was capable of processing lumbar MRI acquired independently of the training and test datasets at a different institution, using different MR scanners, different field strengths, and varying sequence parameters, thereby still providing fair to high diagnostic accuracy.

Although attempts have been made to standardize the nomenclature in the reporting of lumbar spine disease, such as the nomenclature of lumbar discs [25] and the grading of spinal canal stenosis [27], a general reporting and data system for spine MRI is still lacking. In other organ systems, such as the genitourinary tract, the recently established Vesical Imaging Reporting and Data System (VI-RADS) [42,43,44] demonstrated near-perfect interrater agreement in the evaluation of non-muscle-invasive vs. muscle-invasive bladder cancer. However, only moderate intra- and interrater agreement was observed for degenerative conditions of the lumbar spine across different readers, even after adjustment to standardized evaluation criteria [45]. This was mainly attributed to the different weighting of specific disease-defining conditions by the radiologic or surgical observer and may eventually result in limited concordance, even between the same observers over time. Given the potential importance for therapeutic decision making, a more standardized and reproducible approach is therefore still needed to homogenize spine MRI reporting. An automated approach, as we have attempted with CNN-based software, potentially offers the possibility of standardization of spinal MRI findings based on previously defined diagnostic criteria and is not prima vista biased by subjective assessment by the investigator. Because of this, CNNs should not be viewed as the sole basis for reporting, but rather as additional tools that can speed up and facilitate the radiologist’s work and possibly reduce interrater disagreement. Recently, Pacilè et al. investigated the impact of concurrent use of an AI algorithm on the diagnostic performance of radiologists reading mammograms and observed a positive effect on interrater agreement and reading times for cases with low suspicion for malignancy as classified by the CNN [46]. Our results suggest that the CNN we used can especially improve the radiologist’s efficiency for studies that do not show any relevant pathologies, although our study design did not focus on the actual performance of a radiologist using the software. For lumbar spine imaging, no such literature exists, so it will be a subject of further research to explore whether the concurrent use of a CNN can indeed influence the radiologist’s performance.

All of the MR examinations selected for the study at hand could be processed by the software and written reports could be generated, proving the software to be a very reliable tool for the daily practice. The mean processing time for each MRI study was 9:25 min. The preclinical version of the CNN the authors had at their disposal used the computer’s CPU instead of the GPU, resulting in longer processing times. Processing times of approximately 10 min can be acceptable in the clinical setting when the image processing is not done by the radiologist’s workstation simultaneously with the written report, but prior to the radiologist analyzing the images. Still, we see some potential for improvement here to ensure that no delay in the clinical workflow is caused by the algorithm.

## 5. Limitations

We acknowledge several limitations of this study. The retrospective nature generally limits the conclusions to be drawn since the reproducibility of the obtained clinical data cannot be determined. In addition, we are aware of the relatively small sample size. However, considering that many patients admitted to third-level hospitals had already been diagnosed with acute inflammation or malignancy and/or had previously undergone spine surgery, only a limited number of patients could be included for final assessment. Further, the exclusion of patients older than 70 years and with prior history of surgery, trauma, or cancer, does not reflect the clinical reality in most aging societies. However, prior research showed that the mean age is 41 years for lumbar disc herniation and 64 years for spinal canal stenosis, thus the majority of the population affected by lumbar disc herniation and spinal canal stenosis is covered by our study [47]. Our results showed acceptable specificities and sensitivities for the diagnosis of a variety of different degenerative spine pathologies, although the observed PPVs of 13.11–75.00% appear rather low. The main reason for this low PPV is most likely the exclusion of findings by the examining radiologist that were not considered clinically significant, even if they may have been considered abnormal on the basis of objective criteria. This may be considered a limitation of our CNN-based approach, which may not sufficiently reduce the workload of radiologists. However, this will highly depend on the final version of CoLumbo and in how far the developers will succeed in designing a practical user interface to enable the radiologist to evaluate false positive findings as such and flag them as insignificant. For a final assessment of whether the software can positively influence the standardization of findings, reduce reading times, and increase interrater agreement, further studies are necessary and currently pending. The diagnostic objectivity that can result from the machine application of clearly defined diagnostic criteria has the potential to significantly impact the future evaluation of degenerative spine disease and its treatment planning across treatment centers and represents a first milestone in the homogenization of diagnostic reports.

## 6. Conclusions

There is a growing interest in machine-learning algorithms supporting radiologists in their daily work. In the current study, we found that support of diagnosis of various lumbar degenerative changes is feasible with moderate to high diagnostic accuracy using a single comprehensive CNN. Additionally, the high NPVs make this CNN a promising approach to rule out vertebral pathologies in clinical practice. The proposed method paves the way for clinical transition of machine-learning-based diagnostic tools into clinical routine. However, further research is needed to evaluate whether the CNN can influence detection rates, interrater agreement, and reading times of radiologists using the software.

## Figures and Tables

**Table 1:** CNN diagnostic performance. TP: true positive; TN: true negative; FP: false positive; FN: false negative; PPV: positive predictive value; NPV: negative predictive value.

Characteristic	Herniation	Extrusion	Stenosis	Bulging	Nerve Root Compression	Spondylolisthesis
**n**	77 (8.67%)	46 (5.18%)	35 (3.94%)	133 (14.98%)	59 (5.70%)	20 (2.25%)
**TP**	58	41	27	69	42	16
**TN**	713	727	844	602	896	762
**FP**	98	115	9	153	81	106
**FN**	19	5	8	64	17	4
**Sensitivity**	75.33%	89.13%	77.14%	51.88%	71.19%	80.00%
**Specificity**	87.92%	86.34%	98.95%	79.74%	91.71%	87.79%
**Accuracy**	86.82%	86.49%	98.09%	75.56%	90.54%	87.61%
**PPV**	37.18%	26.28%	75.00%	31.08%	34.15%	13.11%
**NPV**	97.40%	99.32%	99.06%	90.39%	98.14%	99.48%
***p***	<0.001	<0.001	1	<0.001	<0.001	<0.001

## Supplementary Materials

The following are available online at https://www.mdpi.com/article/10.3390/diagnostics11050902/s1, Table S1: CNN diagnostic performance at 1.5 T. TP: True positive; TN: True negative; FP: False positive; FN: False negative; PPV: Positive predictive value; NPV: Negative predictive value. Table S2: CNN diagnostic performance at 3 T.

## References

[1] Vos T., Lim S.S., Abbafati C., Abbas K.M., Abbasi M., Abbasifard M., Abbasi-Kangevari M., Abbastabar H., Abd-Allah F., Abdelalim A. (2020). Global burden of 369 diseases and injuries in 204 countries and territories, 1990–2019: A systematic analysis for the Global Burden of Disease Study 2019. Lancet.

[2] Patel N.D., Broderick D.F., Burns J., Deshmukh T.K., Fries I.B., Harvey H.B., Holly L., Hunt C.H., Jagadeesan B.D., Kennedy T.A. (2016). ACR appropriateness criteria low back pain. J. Am. Coll. Radiol..

[3] Rao D., Scuderi G., Scuderi C., Grewal R., Sandhu S.J. (2018). The use of imaging in management of patients with low back pain. J. Clin. Imaging Sci..

[4] McDonald R.J., Schwartz K.M., Eckel L.J., Diehn F.E., Hunt C.H., Bartholmai B.J., Erickson B.J., Kallmes D.F. (2015). The effects of changes in utilization and technological advancements of cross-sectional imaging on radiologist workload. Acad. Radiol..

[5] Jaremko J.L., Poncet P., Ronsky J., Harder J., Dansereau J., Labelle H., Zernicke R.F. (2001). Estimation of spinal deformity in scoliosis from torso surface cross sections. Spine.

[6] Galbusera F., Niemeyer F., Wilke H.-J., Bassani T., Casaroli G., Anania C., Costa F., Brayda-Bruno M., Sconfienza L.M. (2019). Fully automated radiological analysis of spinal disorders and deformities: A deep learning approach. Eur. Spine J..

[7] Klinder T., Ostermann J., Ehm M., Franz A., Kneser R., Lorenz C. (2009). Automated model-based vertebra detection, identification, and segmentation in CT images. Med. Image Anal..

[8] Ma J., Lu L., Zhan Y., Zhou X., Salganicoff M., Krishnan A. (2010). Hierarchical segmentation and identification of thoracic vertebra using learning-based edge detection and coarse-to-fine deformable model. Medical Image Computing and Computer-Assisted Intervention—MICCAI 2010.

[9] Glocker B., Feulner J., Criminisi A., Haynor D.R., Konukoglu E. (2012). Automatic localization and identification of vertebrae in arbitrary field-of-view CT scans. Medical Image Computing and Computer-Assisted Intervention—MICCAI 2012.

[10] Sekuboyina A., Rempfler M., Valentinitsch A., Menze B.H., Kirschke J.S. (2020). Labeling vertebrae with two-dimensional reformations of multidetector CT images: An adversarial approach for incorporating prior knowledge of spine anatomy. Radiol. Artif. Intell..

[11] Peng Z., Zhong J., Wee W., Lee J.-H. (2005). Automated vertebra detection and segmentation from the whole spine MR images. Conf. Proc. IEEE Eng. Med. Biol. Soc..

[12] Han Z., Wei B., Mercado A., Leung S., Li S. (2018). Spine-GAN: Semantic segmentation of multiple spinal structures. Med. Image Anal..

[13] Kim K., Kim S., Lee Y.H., Lee S.H., Lee H.S., Kim S. (2018). Performance of the deep convolutional neural network based magnetic resonance image scoring algorithm for differentiating between tuberculous and pyogenic spondylitis. Sci. Rep..

[14] Kim S., Bae W.C., Masuda K., Chung C.B., Hwang D. (2018). Fine-grain segmentation of the intervertebral discs from MR spine images using deep convolutional neural networks: BSU-Net. Appl. Sci..

[15] Gaonkar B., Beckett J., Villaroman D., Ahn C., Edwards M., Moran S., Attiah M., Babayan D., Ames C., Villablanca J.P. (2019). Quantitative analysis of neural foramina in the lumbar spine: An imaging informatics and machine learning study. Radiol. Artif. Intell..

[16] Zhou Y., Liu Y., Chen Q., Gu G., Sui X. (2018). Automatic lumbar MRI detection and identification based on deep learning. J. Digit. Imaging.

[17] Jamaludin A., Lootus M., Kadir T., Zisserman A., Urban J., Battié M.C., Fairbank J., McCall I. (2017). ISSLS Prize in Bioengineering Science 2017: Automation of reading of radiological features from magnetic resonance images (MRIs) of the lumbar spine without human intervention is comparable with an expert radiologist. Eur. Spine J..

[18] Lundervold A.S., Lundervold A. (2019). An overview of deep learning in medical imaging focusing on MRI. Z. Med. Phys..

[19] Jeon M., Jeong Y.-S. (2020). Compact and accurate scene text detector. Appl. Sci..

[20] Vu T., Nguyen C.V., Pham T.X., Luu T.M., Yoo C.D., Leal-Taixé L., Roth S. (2019). Fast and efficient image quality enhancement via desubpixel convolutional neural networks. Computer Vision—ECCV 2018 Workshops.

[21] Le Cun Y., Bottou L., Bengio Y. Reading checks with multilayer graph transformer networks. Proceedings of the 1997 IEEE International Conference on Acoustics, Speech, and Signal Processing.

[22] Glorot X., Bengio Y. (2010). Understanding the difficulty of training deep feed forward neural networks. J. Mach. Learn. Res..

[23] Pascanu R., Mikolov T., Bengio Y. (2012). On the Difficulty of Training Recurrent Neural Networks. http://arxiv.org/pdf/1211.5063v2.

[24] Up-to-Date Results of the IVDM3Seg Segmentation Challenge. https://ivdm3seg.weebly.com/results.html#.

[25] Fardon D.F., Williams A.L., Dohring E.J., Murtagh F.R., Rothman S.L.G., Sze G.K. (2014). Lumbar disc nomenclature: Version 2.0. Spine J..

[26] Koslosky E., Gendelberg D. (2020). Classification in Brief: The Meyerding classification system of spondylolisthesis. Clin. Orthop. Relat. Res..

[27] Guen Y.L., Joon W.L., Hee S.C., Kyoung-Jin O., Heung S.K., Lee G.Y., Lee J.W., Choi H.S., Oh K.-J., Kang H.S. (2011). A new grading system of lumbar central canal stenosis on MRI: An easy and reliable method. Skelet. Radiol..

[28] Georgiev N., Asenov A. (2019). Automatic segmentation of lumbar spine MRI using ensemble of 2D algorithms. Computational Methods and Clinical Applications for Spine Imaging.

[29] Ronneberger O., Fischer P., Brox T. (2015). U-Net: Convolutional Networks for Biomedical Image Segmentation. http://arxiv.org/pdf/1505.04597v1.

[30] He K., Zhang X., Ren S., Sun J. Deep residual learning for image recognition. Proceedings of the 2016 IEEE Conference on Computer Vision and Pattern Recognition (CVPR).

[31] Lin T.-Y., Maire M., Belongie S., Bourdev L., Girshick R., Hays J., Perona P., Ramanan D., Zitnick C.L., Dollár P. (2014). Microsoft COCO: Common Objects in Context. http://arxiv.org/pdf/1405.0312v3.

[32] Kuhn M. (2008). Building predictive models in R using the Caret Package. J. Stat. Softw..

[33] Cai Y., Osman S., Sharma M., Landis M., Li S. (2015). Multi-modality vertebra recognition in arbitrary views using 3D deformable hierarchical model. IEEE Trans. Med. Imaging.

[34] Law M.W., Tay K., Leung A., Garvin G.J., Li S. (2013). Intervertebral disc segmentation in MR images using anisotropic oriented flux. Med. Image Anal..

[35] Kelm B.M., Wels M., Zhou S.K., Seifert S., Suehling M., Zheng Y., Comaniciu D. (2013). Spine detection in CT and MR using iterated marginal space learning. Med. Image Anal..

[36] Cai Y., Leung S., Warrington J., Pandey S., Shmuilovich O., Li S. (2017). Direct spondylolisthesis identification and measurement in MR/CT using detectors trained by articulated parameterized spine model. Medical Imaging 2017: Image Processing.

[37] Won D., Lee H.-J., Lee S.-J., Park S.H. (2020). Spinal stenosis grading in magnetic resonance imaging using deep convolutional neural networks. Spine.

[38] Park S.H., Han K. (2018). Methodologic guide for evaluating clinical performance and effect of artificial intelligence technology for medical diagnosis and prediction. Radiology.

[39] England J.R., Cheng P. (2019). Artificial intelligence for medical image analysis: A guide for authors and reviewers. Am. J. Roentgenol..

[40] Ting D.S.W., Cheung C.Y.-L., Lim G., Tan G.S.W., Quang N.D., Gan A., Hamzah H., Garcia-Franco R., Yeo I.Y.S., Lee S.Y. (2017). Development and validation of a deep learning system for diabetic retinopathy and related eye diseases using retinal images from multiethnic populations with diabetes. J. Am. Med. Assoc..

[41] Kim D.W., Jang H.Y., Kim K.W., Shin Y., Park S.H. (2019). Design characteristics of studies reporting the performance of artificial intelligence algorithms for diagnostic analysis of medical images: Results from recently published papers. Korean J. Radiol..

[42] Del Giudice F., Barchetti G., De Berardinis E., Pecoraro M., Salvo V., Simone G., Sciarra A., Leonardo C., Gallucci M., Catalano C. (2020). Prospective assessment of vesical imaging reporting and data system (VI-RADS) and its clinical impact on the management of high-risk non–muscle-invasive bladder cancer patients candidate for repeated transurethral resection. Eur. Urol..

[43] Del Giudice F., Leonardo C., Simone G., Pecoraro M., De Berardinis E., Cipollari S., Flammia S., Bicchetti M., Busetto G.M., Chung B.I. (2020). Preoperative detection of vesical imaging-reporting and data system (VI-RADS) score 5 reliably identifies extravesical extension of urothelial carcinoma of the urinary bladder and predicts significant delayed time to cystectomy: Time to reconsider the need for primary deep transurethral resection of bladder tumour in cases of locally advanced disease?. BJU Int..

[44] Del Giudice F., Pecoraro M., Vargas H.A., Cipollari S., De Berardinis E., Bicchetti M., Chung B.I., Catalano C., Narumi Y., Catto J.W.F. (2020). Systematic review and meta-analysis of vesical imaging-reporting and data system (VI-RADS) inter-observer reliability: An added value for muscle invasive bladder cancer detection. Cancers.

[45] Fu M.C., Buerba R.A., Long W.D., Blizzard D.J., Lischuk A.W., Haims A.H., Grauer J.N. (2014). Interrater and intrarater agreements of magnetic resonance imaging findings in the lumbar spine: Significant variability across degenerative conditions. Spine J..

[46] Pacilè S., Lopez J., Chone P., Bertinotti T., Grouin J.M., Fillard P. (2020). improving breast cancer detection accuracy of mammography with the concurrent use of an artificial intelligence tool. Radiol. Artif. Intell..

[47] Cummins J., Lurie J.D., Tosteson T.D., Hanscom B., Abdu W.A., Birkmeyer N.J.O., Herkowitz H., Weinstein J. (2006). Descriptive epidemiology and prior healthcare utilization of patients in the spine patient outcomes research trial’s (SPORT) three observational cohorts: Disc herniation, spinal stenosis, and degenerative spondylolisthesis. Spine.

