# Supplementary material for: Detection of Degenerative Changes on MR Images of the Lumbar Spine with a Convolutional Neural Network: A Feasibility Study
Source: Diagnostics (Basel). 2021 May 19;11(5):902. doi: 10.3390/diagnostics11050902 (PMC8158737; doi:10.3390/diagnostics11050902)
Supplement: Supplementary file 1 [file diagnostics-11-00902-s001.zip › diagnostics-1220537-supplementary.pdf]

diagnostics

MDPI

Supplementary File

Detection of Degenerative Changes on MR Images of the Lumbar Spine with a Convolutional Neural Network: A Feasibility Study

Nils Christian Lehnen [1,2], Robert Haase [1,2], Jennifer Faber [3], Theodor Rüber [4], Hartmut Vatter [5], Alexander Radbruch [1,2] and Frederic Carsten Schmeel [1,2,*]

[1] Department of Neuroradiology, University Hospital Bonn, Rheinische Friedrich-Wilhelms-Universität Bonn, 53127 Bonn, Germany; nils.lehnen@ukbonn.de (N.C.L.); robert.haase@ukbonn.de (R.H.); alexander.radbruch@ukbonn.de (A.R.)
[2] Research Group Clinical Neuroimaging, German Center for Neurodegenerative Diseases, 53127 Bonn, Germany
[3] Department of Neurology, University Hospital Bonn, Rheinische Friedrich-Wilhelms-Universität Bonn, 53127 Bonn, Germany; jennifer.faber@ukbonn.de
[4] Department of Epileptology, University Hospital Bonn, Rheinische Friedrich-Wilhelms-Universität Bonn, 53127 Bonn, Germany; theodor.rueber@ukbonn.de
[5] Department of Neurosurgery, University Hospital Bonn, Rheinische Friedrich-Wilhelms-Universität Bonn, 53127 Bonn, Germany; Hartmut.vatter@ukbonn.de
* Correspondence: Carsten.Schmeel@ukbonn.de; Tel: +49-0228-28716507; Fax. +49-0228-28714321

Citation: Lehnen, N.C.; Haase, R.; Faber, J.; Rüber, T.; Vatter, H.; Radbruch, A.; Schmeel, F.C. Detection of Degenerative Changes on MR Images of the Lumbar Spine with a Convolutional Neural Network: A Feasibility Study. *Diagnostics* **2021**, *11*, 902. https://doi.org/10.3390/diagnostics11050902

Academic Editor: Thomas Baum; Nico Sollmann

Received: 28 April 2021
Accepted: 17 May 2021
Published: 19 May 2021

Publisher's Note: MDPI stays neutral with regard to jurisdictional claims in published maps and institutional affiliations.

Copyright: © 2021 by the authors. Licensee MDPI, Basel, Switzerland. This article is an open access article distributed under the terms and conditions of the Creative Commons Attribution (CC BY) license (http://creativecommons.org/licenses/by/4.0/).

Table S1. CNN diagnostic performance at 1.5T. TP: True positive; TN: True negative; FP: False positive; FN: False negative; PPV: Positive predictive value; NPV: Negative predictive value.

Characteristic	Herniation	Extrusion	Stenosis	Bulging	Nerve Root Compression	Spondylolisthesis
**n**	57 (8.8%)	33 (5.1%)	27 (4.2%)	91 (14.0%)	43 (5.7%)	14 (2.2%)
**TP**	44	29	20	49	28	12
**TN**	514	523	616	444	654	560
**FP**	77	92	5	113	59	74
**FN**	13	4	7	42	15	2
**Sensitivity**	77.19%	87.88%	74.07%	53.85%	65.12%	85.71%
**Specificity**	86.97%	85.04%	99.20%	79.71%	91.73%	88.33%
**Accuracy**	86.11%	85.19%	98.15%	76.08%	90.21%	88.27%
**PPV**	36.36%	23.97%	80.00%	30.25%	32.18%	13.95%
**NPV**	97.53%	99.24%	98.88%	91.36%	97.76%	99.64%
**p**	< 0.001	< 0.001	0.77	< 0.001	< 0.001	< 0.05

Table S2. CNN diagnostic performance at 3T.

Characteristic	Herniation	Extrusion	Stenosis	Bulging	Nerve Root Compression	Spondylolisthesis
**n**	20 (8.3%)	13 (5.4%)	8 (3.3%)	42 (17.5%)	16 (5.7%)	6 (2.5%)
**TP**	14	12	7	20	14	4
**TN**	199	204	228	158	242	202
**FP**	21	92	4	40	22	32
**FN**	6	4	1	22	2	2
**Sensitivity**	70.00%	92.31%	87.50%	47.62%	87.50%	66.67%
**Specificity**	90.46%	89.87%	98.28%	79.80%	91.67%	86.32%
**Accuracy**	88.75%	90.00%	97.92%	74.17%	91.43%	85.83%
**PPV**	40.00%	34.29%	63.64%	33.33%	38.89%	11.11%
**NPV**	97.07	99.51%	99.56%	87.78%	99.18%	99.02%
**p**	< 0.05	< 0.05	0.37	< 0.05	< 0.001	< 0.05
